# A Pin in the Rectum for Thirty Years

**Published:** 1898-09-24

**Authors:** 


### A PIN IN THE RECTUM FOR THIRTY YEARS.

Mr. Dutton Akers records[1] a case in which, so far as the history could be trusted, a pin appears to have been impacted in the rectum for thirty years. He had pain in the rectal region of a pricking character on sitting down, constant desire to defæcate, great pain on so doing, and the motions were small, pipe-like, and streaked with blood. He had frequently sought medical aid, and the treatment adopted had generally taken the form of morphia suppositories or other local applications. There were external hæmorrhoids and considerable induration of the mucous membrane covering the sphincter, above and to the right side of which the head and about half an inch of the body of a pin were to be felt, the head projecting upwards and somewhat across the lumen of the intestine. The removal of the *fons et origo mali* was followed by a rapid subsidence of the symptoms which had existed for so many years.

[1] Lancet, Sept. 10.

